# MicroRNA-148a inhibits the proliferation and promotes the paclitaxel-induced apoptosis of ovarian cancer cells by targeting PDIA3

**DOI:** 10.3892/mmr.2021.12294

**Published:** 2021-07-14

**Authors:** Shuzhen Zhao, Zhengfang Wen, Shanshan Liu, Ying Liu, Xiaorui Li, Yanna Ge, Shaoru Li

Mol Med Rep 12: 3923-3929, 2015; DOI: 10.3892/mmr.2015.3826

Following the publication of this paper, it was drawn to the Editors’ attention by a concerned reader that the cell apoptosis assay data shown in Figs. 1C and 4D were strikingly similar to data appearing in different form in other articles by different authors. Owing to the fact that the contentious data in the above article had already been published elsewhere, or were already under consideration for publication, prior to its submission to *Molecular Medicine Reports*, the Editor has decided that this paper should be retracted from the Journal. The authors were asked for an explanation to account for these concerns, but the Editorial Office did not receive any reply. The Editor apologizes to the readership for any inconvenience caused.

